# Supplementary material for: Renin Feedback Is an Independent Predictor of Outcome in HFpEF
Source: J Pers Med. 2021 May 3;11(5):370. doi: 10.3390/jpm11050370 (PMC8147649; doi:10.3390/jpm11050370)
Supplement: Supplementary file 1 [file jpm-11-00370-s001.zip › jpm-1190699-supplementary.pdf]

Supplementary materials

Supplementary table 1- Renin angiotensin aldosterone system (RAAS) profiles in patients with and without angiotensin conversion enzyme inhibitor (ACEi) therapy.

RAAS Triple-A Analysis	ACEi (n=45)	No ACEi (n=105)	p-value
Angiotensin I, pM	329.9 (149.3-916.5)	127.4 (33.6-519.5)	0.002
Angiotensin II, pM	58.7 (9.6-117.4)	198.2 (73.6-833.9)	<0.001
Aldosterone, pM	136.8 (59.4-303.9)	145.5 (63.5-241.1)	0.977
AA2-Ratio pM/pM	2.4 (1.1-8.1)	0.7 (0.2-2.1)	<0.001
PRA-S, pM	412.4 (215.0-1119.5)	358.7 (113.8-1255.9)	0.406
ACE-S, pM/pM	0.1 (0.1-0.3)	1.8 (1.3-2.3)	<0.001

All values are given as median and interquartile ranges.
AA2 indicates Angiotensin-Aldosterone 2 ratio; PRA, plasma renin activity and ACE, angiotensin converting enzyme.

Supplementary table 2- Renin angiotensin aldosterone system (RAAS) profiles in patients with and without angiotensin receptor blocker (ARB) therapy.

RAAS Triple-A Analysis	ARB (n=66)	No ARB (n=84)	p-value
Angiotensin I, pmol/L	131.7 (45.2-654.6)	264.6 (61.6-64.9)	0.187
Angiotensin II, pmol/L	245.4 (90.6-873.2)	93.8 (26.7-277.1)	<0.001
Aldosterone, pmol/L	106.4 (59.3-221.6)	153.2 (70.0-313.3)	0.137
AA2-Ratio, (pmol/L)/(pmol/L)	0.4 (0.2-1.4)	1.5 (0.6-4.3)	<0.001
PRA-S, pmol/L	388.8 (130.9-1533.7)	334.8 (147.9-1071.8)	0.822
ACE-S, (pmol)/(pmol/L)	1.8 (1.4-2.2)	0.7 (0.1-1.8)	<0.001

Values are given as median and interquartile ranges.
AA2 indicates Angiotensin-Aldosterone 2 ratio; PRA, plasma renin activity and ACE, angiotensin converting enzyme.

Supplementary table 3- Renin angiotensin aldosterone system (RAAS) profiles in patients with and without treatment with RAAS-inhibitors (RAASi) at baseline.

RAAS Triple-A Analysis	RAASi (n=111)	No RAASi (n=39)	p-value
Angiotensin I, pmol/L	223.6 (65.6-776.5)	117.7 (24.5-479.1)	0.084
Angiotensin II, pmol/L	119.8 (34.1-425.9)	119.3 (53.1-739.4)	0.417

Aldosterone, pmol/L	124.1 (59.3-241.1)	177.2 (94.3-338.6)	0.087
AA2 ratio (pmol/L)/(pmol/L)	1.0 (0.2-3.5)	0.9 (0.4-2.4)	0.973
PRA-S, pmol	213.8 (96.5-1024.0)	213.8 (96.5-1024.0)	0.262
ACE-S, (pmol/L)/(pmol/L)	1.7 (1.0-2.6)	1.7 (1.0-2.6)	0.010

All values are given as median and interquartile ranges.
AA2 indicates aldosterone/angiotensin II ratio; PRA-S, plasma renin activity surrogate; ACE-S, angiotensin converting enzyme surrogate and ACE2-S, angiotensin converting enzyme 2 surrogate.

Supplementary table 4- Renin angiotensin aldosterone system (RAAS) profiles in patients treated with angiotensin converting enzyme inhibitors (ACEi) versus patients treated with angiotensin receptor blockers (ARB).

RAAS Triple-A Analysis	ACEi (n=45)	ARB (n=66)	p-value
Angiotensin I, pmol/L	329.9 (149.3-916.5)	131.7 (45.2-654.6)	0.007
Angiotensin II, pmol/L	58.7 (9.6-117.4)	245.4 (90.6-873.2)	<0.001
Aldosterone, pmol/L	136.8 (59.4-303.9)	106.4 (59.3-221.6)	0.509
AA2-Ratio (pmol/L)/(pmol/L)	2.4 (1.1-8.1)	0.4 (0.2-1.4)	<0.001
PRA -S, pmol/L	412.4 (215.0-1119.5)	388.8 (130.9-1533.7)	0.714
ACE-S, (pmol/L)/(pmol/L)	0.1 (0.1-0.3)	1.8 (1.4-2.2)	<0.001

All values are given as median and interquartile ranges.
AA2-Ratio indicates aldosterone/angiotensin II ratio; PRA-S, plasma renin activity; ACE-S, angiotensin converting enzyme activity.

Supplementary table 5- Renin angiotensin aldosterone system (RAAS) profiles in patients treated with mineralocorticoid receptor antagonists (MRA) at time at baseline versus those who were not treated with MRA.

RAAS Triple-A Analysis	MRA (n=53)	No MRA (n=97)	p-value
Angiotensin I, pmol /L	346.3 (161.9-851.4))	105.4 (35.3-409.0)	<0.001
Angiotensin II, pmol/L	328.8 (74.8-1099.2)	95.0 (34.1-242.8)	0.001

**Aldosterone**, pmol/L	239.0 (102.5-489.5)	108.2 (51.7-200.62	<0.001
AA2-Ratio (pmol/L)/(pmol/L)	0.8 (0.2-2.1)	1.1 (0.3-3.3)	0.342
**PRA -S**, pmol/L	927.9 (271.3-1658.9)	222.0 (113.8-702.3)	<0.001
ACE-S, (pmol/L)/(pmol/L)	1.4 (0.3-2.0)	1.6 (0.4-2.2)	0.611

AA2-Ratio indicates aldosterone/angiotensin II ratio; PRA-S, plasma renin activity; ACE-S, angiotensin converting enzyme activity.

Supplementary figure 1- CONSORT diagram of the studied population.

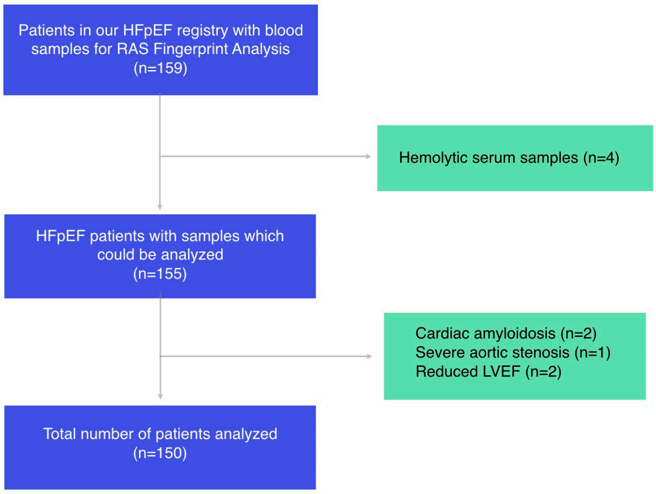

HFpEF indicates heart failure with preserved ejection fraction; RAS, renin angiotensin aldosterone system; LVEF, left ventricular ejection fraction
